# Identification and Targeting of an Interaction between a Tyrosine Motif within Hepatitis C Virus Core Protein and AP2M1 Essential for Viral Assembly

**DOI:** 10.1371/journal.ppat.1002845

**Published:** 2012-08-16

**Authors:** Gregory Neveu, Rina Barouch-Bentov, Amotz Ziv-Av, Doron Gerber, Yves Jacob, Shirit Einav

**Affiliations:** 1 Department of Medicine, Division of Infectious Diseases and Geographic Medicine, and Department of Microbiology and Immunology, Stanford University School of Medicine, Stanford, California, United States of America; 2 The Mina and Everard Goodman Faculty of Life Sciences and The Nanotechnology Institute, Bar-Ilan University, Ramat-Gan, Israel; 3 Department of Virology, Unité de Génétique, Papillomavirus et Cancer Humain (GPCH), Pasteur Institute, Paris, France; University of California, San Diego, United States of America

## Abstract

Novel therapies are urgently needed against hepatitis C virus infection (HCV), a major global health problem. The current model of infectious virus production suggests that HCV virions are assembled on or near the surface of lipid droplets, acquire their envelope at the ER, and egress through the secretory pathway. The mechanisms of HCV assembly and particularly the role of viral-host protein-protein interactions in mediating this process are, however, poorly understood. We identified a conserved heretofore unrecognized YXXΦ motif (Φ is a bulky hydrophobic residue) within the core protein. This motif is homologous to sorting signals within host cargo proteins known to mediate binding of AP2M1, the μ subunit of clathrin adaptor protein complex 2 (AP-2), and intracellular trafficking. Using microfluidics affinity analysis, protein-fragment complementation assays, and co-immunoprecipitations in infected cells, we show that this motif mediates core binding to AP2M1. YXXΦ mutations, silencing AP2M1 expression or overexpressing a dominant negative AP2M1 mutant had no effect on HCV RNA replication, however, they dramatically inhibited intra- and extracellular infectivity, consistent with a defect in viral assembly. Quantitative confocal immunofluorescence analysis revealed that core's YXXΦ motif mediates recruitment of AP2M1 to lipid droplets and that the observed defect in HCV assembly following disruption of core-AP2M1 binding correlates with accumulation of core on lipid droplets, reduced core colocalization with E2 and reduced core localization to *trans*-Golgi network (TGN), the presumed site of viral particles maturation. Furthermore, AAK1 and GAK, serine/threonine kinases known to stimulate binding of AP2M1 to host cargo proteins, regulate core-AP2M1 binding and are essential for HCV assembly. Last, approved anti-cancer drugs that inhibit AAK1 or GAK not only disrupt core-AP2M1 binding, but also significantly inhibit HCV assembly and infectious virus production. These results validate viral-host interactions essential for HCV assembly and yield compounds for pharmaceutical development.

## Author Summary

Novel antiviral strategies are needed to combat the HCV pandemic. Understanding viral-host determinants involved in mediating assembly is critical for the development of drugs targeting this stage of the HCV life cycle. We identified a conserved heretofore unrecognized tyrosine motif within core, which is homologous to sorting signals within host cargo proteins that are recognized by AP2M1, a membrane trafficking protein. By combining novel proteomic techniques with molecular virology, RNAi, dominant-interfering, and pharmacological approaches, we show that this motif mediates core binding to AP2M1 and HCV assembly and that AP2M1 is essential for HCV assembly. Core's tryosine motif recruits AP2M1 to lipid droplets, the site of viral assembly, and disruption of core-AP2M1 binding alters core's sub-cellular localization and colocalization with the envelope protein E2. AAK1 and GAK, host kinases that stimulate AP2M1's interactions with cargo proteins, regulate core-AP2M1 binding and are essential for HCV assembly. Last, we discovered kinase inhibitors, which bind AAK1 or GAK and disrupt core-AP2M1 binding and HCV assembly. Our study provides insight into mechanisms of HCV assembly and yields candidate targets and compounds for pharmaceutical development. Since some of the discovered compounds are approved anti-cancer drugs, there may be an opportunity for repurposing them as antivirals.

## Introduction

HCV is a major cause of liver disease, estimated to infect 170 million people worldwide [1]. Although combining interferon-ribavirin-based regimens with HCV protease or polymerase inhibitors improves response rates, resistance and toxicity continue to pose major challenges [2]. A “cocktail” of drugs, each targeting an independent function will likely offer the best pharmacological control. There is therefore an urgent need for drugs directed at novel targets.

HCV is a positive, single-stranded RNA virus from the Flaviviridae family. Its 9.6 kb genome encodes a single polyprotein, which is proteolytically cleaved into three structural proteins (core and the glycoproteins, E1 and E2) and seven non-structural (NS) proteins (p7, NS2, NS3, NS4A, NS4B, NS5A, NS5B) [3], [4], [5].

Studies of HCV assembly have become possible only after the recent establishment of a complete HCV replication system in cell culture [6]. This stage of the viral life cycle thus remains poorly understood. The current model for infectious virus production is that HCV particles are assembled on the surface of lipid droplets (LD) or at the ER in juxtaposition to LD [7], [8], [9], where the core protein is concentrated [10], [11], [12], [13], [14], [15]. While not definitively proven, similarly to flaviviruses ([16], reviewed in [17]), HCV envelopment is believed to occur at an ER-derived compartment [10], [18], [19], where the envelope proteins are retained [20], [21]. Unlike flaviviruses, HCV particles become infectious upon envelopment. Viral particles then exit the cell via the secretory pathway [18], [19], where they co-traffic with various components of ER, TGN, and recycling endosomes [22]. Since interactions of very low-density lipoprotein (VLDL) components, such as apolipoprotein E, with HCV are essential for infectious virus production, it is thought that HCV particles are associated with or internalized in VLDL structures [23], [24], [25], [26], [27].

Assembly requires a coordinated integration of cellular and viral factors that bring together the core (capsid) protein with the E1 and E2 envelope proteins, and the viral RNA (reviewed in [19], [28]). Maturation of the core protein is achieved through cleavage of its carboxy terminus (aa 175–191) to give a 174 aa dimeric membrane protein composed of two domains, D1 (aa 1–117) and D2 (aa 118–174) (reviewed in [29]). Mature core is directed from the ER membrane onto the surface of LDs [30], [31], [32]. DGAT1, an enzyme essential for LD biogenesis, was shown to bind core and to mediate its localization to the assembly sites at or near LD [33]. Additional host factors, such HSC70 [34], ANXA2 [35], and the late endocytic protein, HRS [36], as well as several NS proteins, including p7, NS2, NS3, and NS5A, have been shown to be essential for HCV assembly [37], [38], [39], [40], [41], [42], [43], [44], [45], [46]. Nevertheless, a mechanistic understanding of HCV assembly following recruitment of core to LD is far from being complete. In particular, interactions between specific viral protein determinants and cellular factors involved in mediating this stage of viral assembly, have not been reported.

**Figure 1:**
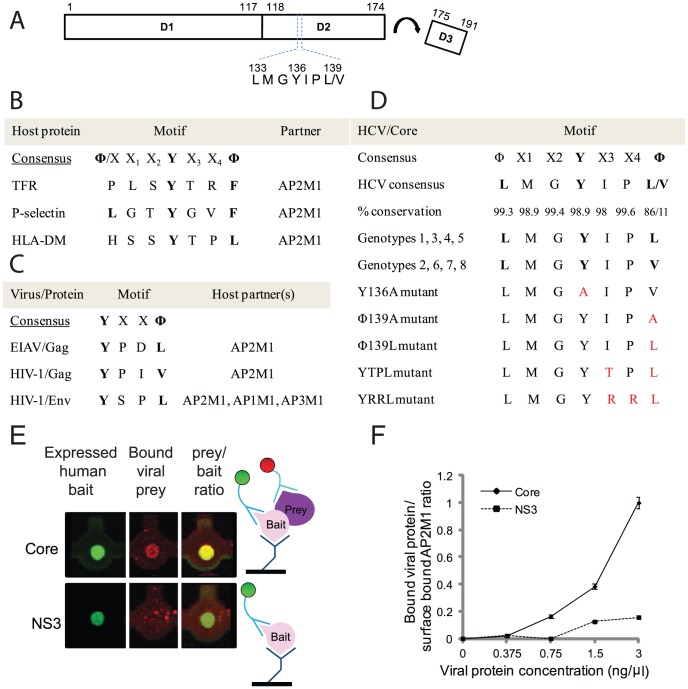
Core harbors a YXXΦ motif and binds AP2M1. A. Schematics of core. The location of the identified motif is indicated. B–C. Consensus sequences of YXXΦ motifs from representative human (B) and viral (C) proteins. D. The consensus sequence of all HCV isolates, clones used in this study, and engineered core mutants. E. Fluorescent images from a microfluidic chip and schematics. Left: AP2M1-V5-his was anchored to the device surface via its interaction with anti-His antibodies and labeled with anti-V5-FITC antibodies. Middle: T7-tagged core or NS3 were incubated with surface bound AP2M1 and labeled with anti-T7-Cy3 antibodies. Interactions were trapped mechanically by MITOMI. Cy3 signal representing bound viral protein is shown following a wash. Right: an overlay of the Cy3 and FITC signals, representing bound viral prey to human bait ratio. F. *In vitro* binding curves of core-T7 and NS3-T7 to surface bound AP2M1. Y axis represents bound viral protein to surface bound AP2M1 ratio.

Cellular membrane trafficking processes depend to a large extent on the function of tyrosine-based sorting signals. YXXΦ motifs (Φ is a bulky hydrophobic residue - L/I/M/V/F) within membrane host cargo proteins are recognized by the μ subunit of clathrin adaptor protein (AP) complexes (Figure 1B) [47], [48], [49]. AP complexes are heterotetramers that mediate the sorting of cargo proteins to specific membrane compartments within the cell [49]. Four adaptor protein complexes have been described in mammalian cells, designated AP-1 through AP-4. The four subunits of an AP complex contain two large (a *β* and one of either an *α*, *γ*, *δ*, or *ε*) subunits (110–130 kDa), a medium (*μ*) subunit (approximately 50 kDa), and a small (*σ*) subunit (15–20 kDa) [49]. Each of the AP complexes is involved in distinct trafficking pathways. AP2M1, the μ subunit of AP-2, is known to mediate sorting of cargo proteins harboring YXXΦ or dileucine motifs into clathrin-coated vesicles (CCV), that either form at the cell surface and are destined for fusion with early endosomes [49] or traffic in lysosomal transport pathways [50]. AP2M1 activity is regulated by two host cell kinases, adaptor-associated kinase 1 (AAK1) and cyclin G-associated kinase (GAK). Phosphorylation of a T156 residue in AP2M1 by these kinases is known to stimulate its binding to cargo proteins [51], [52], [53]. Recognition of YXXΦ signals within retroviral structural proteins by AP2M1 has been shown to be involved in mediating intracellular trafficking of the Gag protein and infectious virus production [54], [55], [56], [57], [58] (Figure 1C). No equivalent signals have been previously described in Flaviviridae proteins. Furthermore, while late endocytic proteins have been shown to be involved in viral egress [59], [60], [61], a role of early endocytic functions in the production of infectious HCV particles has not been described.

We have recently identified a heretofore unrecognized conserved YXXΦ motif within the HCV core protein. We hypothesized that core's YXXΦ motif may mediate binding to AP2M1, that this interaction might be critical for the late stages of HCV infection, and that this knowledge could be translated into the development of novel antiviral strategies. Here we show that the YXXΦ motif mediates core binding to AP2M1. Either core YXXΦ mutations, overexpression of a dominant negative AP2M1 mutant or silencing of AP2M1, AAK1 or GAK expression inhibited HCV assembly. AP2M1 significantly localized to LD in cells overexpressing core or infected with HCV, but not in naive cells. A YXXΦ mutation decreased AP2M1 recruitment to LD, and like AP2M1 knockdown, was associated with increased core localization to LD and significant reduction of its colocalization with a TGN marker and with the E2 envelope protein. Last, approved anti-cancer drugs that inhibit AAK1 or GAK, dramatically inhibited core-AP2M1 binding, HCV assembly, and infectious virus production.

## Results

### Identification of a YXXΦ motif within HCV core

Inspection of the primary sequence of the HCV core protein reveals a conserved YIP(V/L) motif within the second domain (D2) of the protein (Figures 1A, D). This motif conforms to the YXXΦ sorting signal consensus recognized by AP2M1 [47], [48], [49].

### Core binds AP2M1

Interactions of sorting signals with clathrin adaptors and endocytic components are typically weak (Kd of binding at a µM range), transient [48], [62], and involve membrane proteins, thus difficult to study by standard technologies [63], [64]. To determine whether HCV core binds AP2M1, we therefore used proteomic platforms that overcome these challenges. *In vitro* microfluidics affinity assays are based on mechanical trapping of molecular interactions (MITOMI), which eliminates the off-rate problem facing current platforms, and thus allows studying weak and transient interactions, with nanoliter protein consumption [65], [66], [67]. We used a microfluidics format that enables a high fidelity analysis of protein-protein interactions (P-PIs) [67]. *In vitro* protein expression in the presence of microsomal membranes and binding experiments with MITOMI were performed essentially as described [65], [66], [67] (Figure 1E and Figure S1 in Text S1). These assays detected binding of AP2M1 to core. The degree of binding correlated with increasing core concentration (Figure 1F). Background binding of AP2M1 to a control HCV protein, NS3, was 4–20 fold lower and did not increase significantly with increased protein concentration.

**Figure 2:**
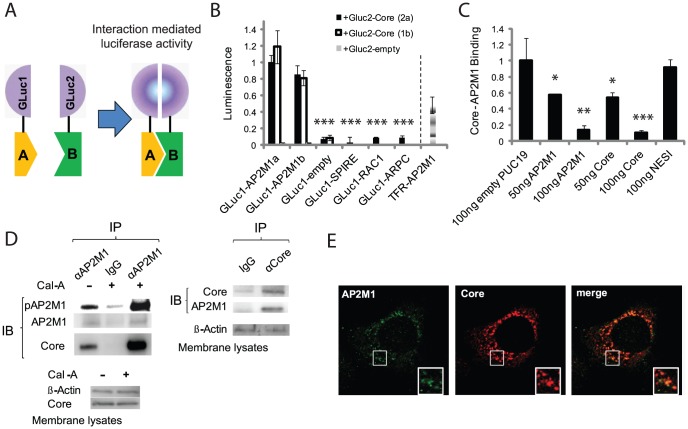
Core binds AP2M1 in cells and in the context of HCV infection. A. Schematics of the PCAs format. A and B represent prey and bait proteins and GLuc1/2 are fragments of *Gaussia* luciferase. B. Cells were cotransfected with combinations of plasmids indicated below the graph with those indicated in the legend. Y axis represents luminescence relative to the core-AP2M1 signal. The banded bar on the right represents AP2M1 binding to the host cargo protein TFR. C. Core-AP2M1 binding in the presence of free AP2M1, core or NESI. Y axis represents luminescence ratio (the average luminescence signal detected in cells transfected with Gluc1-AP2M1 and Gluc2-core divided by the average of luminescence measured in control wells transfected with Gluc1-AP2M1 and an empty Gluc2 vector with those transfected with Gluc2-core and an empty Gluc1 vector) relative to core-AP2M1 binding in the presence of empty PUC19. D. Immunoprecipitations (IPs) in membranes of HCV infected cells. Left panels: Anti-AP2M1 antibodies or IgG were used for IP. Membranes were immunoblotted (IB) with anti-phospho-AP2M1, anti-AP2M1, anti-core, and anti-actin antibodies. Cal-A represents calyculin-A. Right panels: Anti-core antibodies or IgG were used for IP. Membranes were immunoblotted (IB) with anti-core, anti-AP2M1, and anti-actin antibodies. E. Representative confocal IF microscopy images of AP2M1 and core in Huh-7.5 cells 3 days postelectroporation with J6/JFH HCV RNA. Data represent means±s.d. (error bars) from three independent experiments in triplicates (n>20 in E). * p<0.05, ** p<0.01, *** p<0.001.

To determine whether the core-AP2M1 interaction occurs in cells, we used protein-fragment complementation assays (PCAs) based on reversible reconstitution of a *Gaussia* princeps luciferase reporter (Figure 2A). The current format was optimized for improved signal, thus providing a highly sensitive means for measuring challenging P-PIs [68]. Significant luciferase signal was detected in Huh-7.5 cells cotransfected with plasmids encoding the two reporter fragments fused to the prey and bait proteins (GLuc1-AP2M1 and GLuc2-core). Background levels of binding were detected in cells cotransfected with either the GLuc1-AP2M1 or GLuc2-core constructs and the empty reciprocal vector, the two empty GLuc vectors, or GLuc1 fused to three unrelated proteins (SPIRE, RAC1, and ARPC). The apparent affinity of AP2M1 to core was higher than to transferrin receptor (TFR), a host cargo protein harboring a YXXΦ signal, known to be recognized by AP2M1 [69] (Figure 2B). Binding was not genotype-specific, as core proteins derived from either the 2a [6] or 1b [70] genotypes demonstrated comparable levels of AP2M1 binding. Furthermore, there were no significant differences in core binding to the two isoforms of AP2M1(a/b) (Figure 2B). Comparable results were demonstrated in Huh-7.5 (human hepatoma derived) cells, representing the most relevant cell model (Figure 2B), and 293T cells (data not shown). Binding appeared specific, as increasing concentrations of free core or AP2M1, but not nuclear export signal-interacting protein (NESI), a control protein essential for mediating hepatitis D virus assembly [71], progressively decreased core-AP2M1 binding (Figure 2C).

To determine whether core binds AP2M1 in the context of authentic HCV infection, we performed co-immunoprecipitation assays in membrane fractions prepared from Huh-7.5 cells infected with cell culture-grown HCV (J6/JFH) [6]. AP2M1 could bring down core when anti-AP2M1 antibodies but not IgG controls were added to the membrane lysates. Binding was significantly augmented by calyculin A (an inhibitor of AP2M1 dephosphorylation, which “locks” AP2M1 in its YXXΦ binding active conformation [72]) (Figure 2D). Binding in reciprocal conditions was similarly demonstrated (Figure 2D). We also investigated colocalization of core with AP2M1 in Huh-7.5 cells 72 hr following electroporation with J6/JFH HCV RNA. Quantitative confocal immunofluorescence (IF) analysis revealed extensive colocalization of core and AP2M1 in these cells (with 67±6% colocalization of AP2M1 stained puncta with core) (Figure 2E).

### Core's YXXΦ motif is essential for AP2M1 binding and HCV assembly

**Figure 3:**
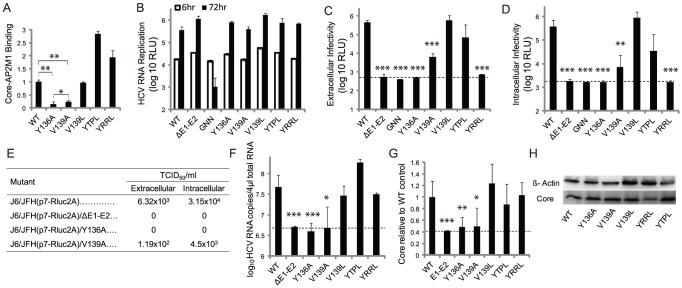
Core's YXXΦ motif mediates AP2M1 binding and viral assembly and is functionally interchangeable with other YXXΦ signals. A. Binding of wild type (WT) and core mutants to AP2M1 by PCAs. Y axis represents luminescence ratio (the average luminescence signal detected in cells transfected with Gluc1-AP2M1 and the various forms of Gluc2-core divided by the average of luminescence measured in control wells transfected with Gluc1-AP2M1 and an empty Gluc2 vector with those transfected with the respective Gluc2-core and an empty Gluc1 vector) relative to WT core-AP2M1 binding. B. HCV RNA replication by *Renilla* luciferase assays at 6 hr (white) and 72 hr (black) postelectroporation with J6/JFH(p7-Rluc2A) harboring the corresponding mutations. ΔE1–E2 is an assembly defective control. GNN is a replication defective polymerase mutant. C. Extracellular infectivity by luciferase assays in naive Huh-7.5 cells infected with supernatants derived from the electroporated cells. D. Intracellular infectivity by luciferase assays in naive Huh-7.5 cells infected with clarified cell lysates derived from the electroporated cells. E. Intra- and extracellular infectivity titers measured by limiting dilution assays. TCID_50_ is 50% tissue culture infectious dose. F. Viral RNA release into the culture supernatant at 72 hr postelectroporation measured by qRT-PCR. G. HCV core protein release into the culture supernatant at 72 hr postelectroporation determined by ELISA relative to WT control. H. Levels of core protein by Western analysis in lysates prepared from cells infected with virus harboring the corresponding mutations. Means and s.d. (error bars) of results from at least three independent experiments in triplicates are shown. The dashed horizontal lines represent background levels of luciferase activity. RLU is relative light units. * p<0.05, ** p<0.01, *** p<0.001.

Using a series of point mutations (Figure 1D), we tested whether AP2M1 binding is mediated by core's YXXΦ motif. A Y136A core mutation reduced AP2M1 binding measured by PCAs by ∼10 fold compared with wild type (WT) core, whereas a V(Φ)139A mutation caused less inhibition of binding (Figure 3A). To study the role of core's YXXΦ motif in HCV infection, these mutations were introduced into the J6/JFH(p7-Rluc2A) HCV genome - a *Renilla* luciferase-containing reporter virus that replicates and produces high titers of virus in Huh-7.5 cells [73]. Cells were electroporated with *in vitro* transcribed RNA generated from each construct. HCV RNA replication of these viral mutants was comparable to that of the WT virus, as measured by luciferase reporter gene-linked assays (Figure 3B), and qRT-PCR (Figure S2 in Text S1). In contrast, a polymerase-defective mutant, J6/JFH(p7-Rluc2A)-GNN, did not replicate. Luciferase assays in naive cells inoculated with supernatants derived from cells electroporated with viral genome harboring the Y136A core mutation measured undetectable levels of extracellular infectivity. The V139A mutation decreased infectivity by ∼1.5 logs compared to WT virus (Figure 3C). Intracellular infectivity, measured in naive cells infected with clarified supernatants derived from lysed electroporated cells, mirrored the diminished extracellular infectivity (Figure 3D), suggesting that core's YXXΦ motif mediates virions assembly and not release. Essentially no infectious virus was produced either intra- or extracellularly by assembly (ΔE1–E2) or replication (GNN) defective controls. Infectivity titers of WT virus measured by limiting dilution assays were comparable to those previously reported with this reporter system [74]. Consistent with the luciferase assays described above, while the V139A core mutation decreased the extra- and intracellular infectivity titers by a ∼1–1.5 log compared with WT virus, an undetectable level of infectivity titers was measured with virus harboring the Y136A core mutation or E1–E2 deletion (Figure 3E). The effect of core mutations on infectivity correlated with their effect on AP2M1 binding. To exclude the possibility that core's mutations affected infectivity by causing particle disassembly or production of defective core protein- and RNA-containing particles, we determined production of noninfectious particles. Detectable levels of HCV RNA and core protein release were measured in supernatants of cells harboring replicating genomes by qRT-PCR and ELISA assays, respectively, as described [73], [74](Figures 3F, 3G). Nevertheless, the levels released by the Y136A and V139A core mutants were not significantly higher than those released by the assembly-defective ΔE1–E2 mutant, suggesting that noninfectious particles were not produced. Core expression was not affected by the mutations, as Western analysis (Figure 3H) and fluorescence microscopy (data is not shown) demonstrated protein expression at WT levels. Reversion of the infectivity phenotype was detected by luciferase assays in Huh-7.5 cells infected with HCV harboring the Y136A and V139A core mutations following two weeks of passaging. This reversion coincided with the emergence of primary-site revertants by sequencing analysis. These results provide additional evidence for the requirement of maintaining a functional YXXΦ motif for supporting HCV replication.

Together, these data suggest that the AP2M1 binding motif within core is required for viral assembly *in vitro*.

### Core's YXXΦ motif is functionally interchangeable with other YXXΦ sorting signals

To determine whether core's YXXΦ motif is functionally interchangeable with homologous signals, we introduced a V139L mutation, thus “swapping” the genotype 2a core's sequence with that of genotype 1b. Similarly, the YTPL and YRRL sequences, known to mediate binding of HLA-DM [75] and thrombopoietin receptor [76] to AP2M1, respectively, were used to substitute the core's YXXΦ sequence (Figure 1D). Binding of core harboring these sequences to AP2M1 was either comparable to or greater than that of WT core, as determined by PCAs (Figure 3A). These mutations had no effect on HCV RNA replication (Figure 3B, Figure S2 in Text S1). In correlation with their biochemical phenotype, the intracellular and extracellular infectivity of the V139L and YTPL core mutants were comparable to that of WT virus (Figures 3C, 3D). This functional interchangeability supports that core's YXXΦ motifs exerts its function via interactions with host cell proteins. Despite its efficient binding to AP2M1 (Figure 3A) and stability by Western analysis (Figure 3H), the YRRL mutant did not produce detectable levels of infectious virus (Figures 3C, 3D). Interestingly, this mutant released HCV RNA and core protein into supernatants of electroporated cells at levels comparable to that of WT core, likely reflecting production of noninfectious particles (Figures 3F, 3G). The YRRL mutant may thus impact other function of core in infectious virus production that is independent of its binding to AP2M1.

### AP2M1 is essential for HCV assembly

**Figure 4:**
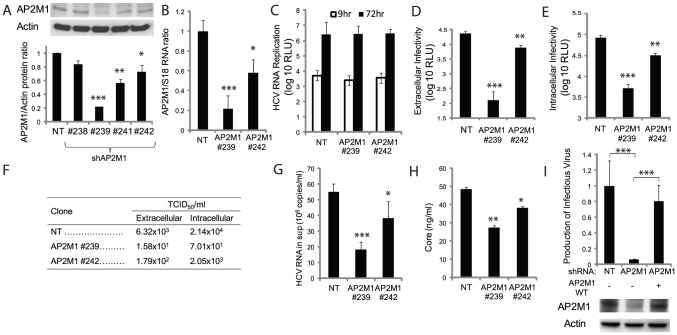
AP2M1 depletion inhibits HCV assembly. A. AP2M1 protein levels by quantitative Western analysis in stable clones harboring shRNA lentiviral constructs targeting the AP2M1 gene and a non-targeting (NT) sequence. A representative membrane and combined data from three independent measurements are shown. Y axis represents AP2M1 to actin protein ratio relative to NT control. B. AP2M1/S18 RNA ratio by qRT-PCR in selected stable clones relative to NT control. C. The indicated clones were electroporated with J6/JFH(p7-Rluc2A). HCV RNA replication in these clones by luciferase assays at 9 hr (white) and 72 hr (black) postelectroporation. D. Extracellular infectivity measured by luciferase assays in naive cells inoculated with supernatants derived from the various stable cell clones. E. Intracellular infectivity by luciferase assays in naive Huh-7.5 cells infected with clarified cell lysates derived from the electroporated cells. F. Intra- and extracellular infectivity titers measured by limiting dilution assays. TCID_50_ is 50% tissue culture infectious dose. G. Viral RNA release into the culture supernatant at 72 hr postelectroporation measured by qRT-PCR. H. HCV core protein release into the culture supernatant at 72 hr postelectroporation, as determined by ELISA. I. Infectious virus production relative to NT control (top panel) and levels of AP2M1 protein by a Western blot analysis (bottom panel) in cells concurrently transduced with lentiviruses expressing shAP2M1 and shRNA resistant WT AP2M1 cDNA (AP2M1-WT). Means and s.d. (error bars) of results from at least three independent experiments are shown. RLU is relative light units. * p<0.05, ** p<0.01, *** p<0.001.

We then determined the functional relevance of AP2M1 to the HCV life cycle. Stable Huh-7.5 clones harboring short hairpin RNA (shRNA) lentiviral constructs targeting distinct regions in the AP2M1 gene or a non-targeting (NT) sequence were established. Effective suppression of AP2M1 levels was achieved (Figures 4A, 4B), without apparent cytostatic or cytotoxic effects. The effect of AP2M1 depletion on infectious virus production was studied in these clones following electroporation with J6/JFH(p7-Rluc2A) RNA. AP2M1 knockdown had no effect on HCV RNA replication as measured in these stable clones by luciferase assays (Figure 4C) and qRT-PCR 72 hr following electroporation (Figure S3 in Text S1). Supernatants collected at 72 hr postelectroporation were used to inoculate naive Huh-7.5 cells followed by luciferase assays at 72 hr postinoculation. As shown in Figure 4D, AP2M1 depletion reduced extracellular infectivity by >2 logs compared with NT control. Intracellular infectivity, measured in naive cells infected with clarified supernatants derived from lysed electroporated cells, mirrored the diminished extracellular infectivity(Figure 4E) and correlated with the degree of AP2M1 depletion. Measurements of intra- or extracellular infectivity titers by limiting dilution assays demonstrated consistent results (Figure 4F). AP2M1 depletion did not increase production of noninfectious particles, as suggested by the levels of HCV RNA and core protein release measured in supernatants of cells by qRT-PCR and ELISA assays, respectively (Figures 4G, 4H). Similar effects on infectious virus production were demonstrated in Huh-7.5 cells transiently depleted for AP2M1 by siRNAs and either electroporated with the J6/JFH(p7-Rluc2A) RNA or infected with culture grown J6/JFH virus (titer: 1.2×10^5^ TCID_50_/ml) [6] (Figure S4 in Text S1). The effects of silencing endogenous AP2M1 on infectious virus production were rescued by ectopic expression of shRNA-resistant WT AP2M1 harboring a wobble mutation within the site targeted by the shRNA, largely excluding the possibility of off-target effects causing the observed phenotype (Figure 4I). Our stable and transient RNAi approaches thus both suggest that AP2M1 is essential for efficient HCV assembly.

### Disruption of core-AP2M1 binding abolishes recruitment of AP2M1 to LD, alters the sub-cellular localization of core, and core colocalization with E2

**Figure 5:**
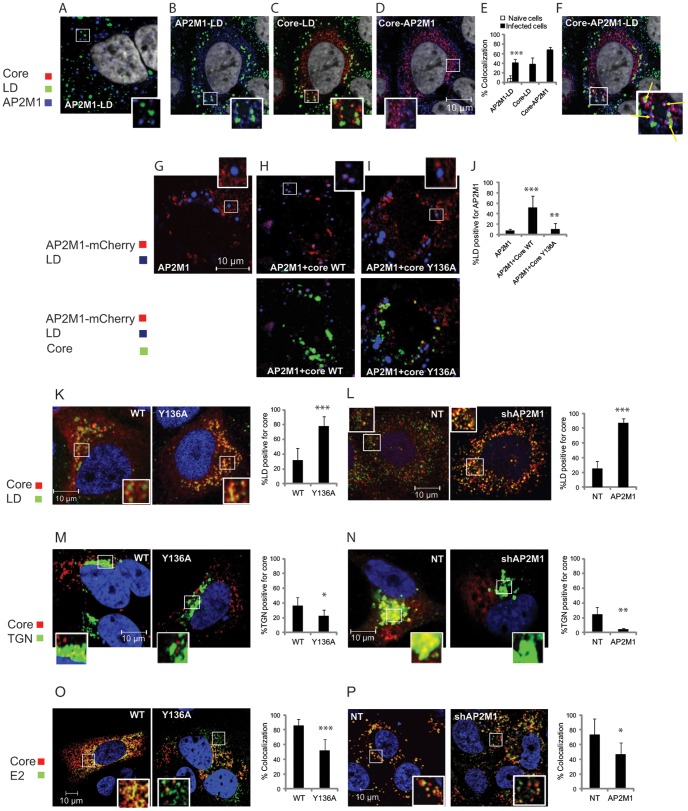
Disruption of core-AP2M1 binding abolishes recruitment of AP2M1 to LD and alters the sub-cellular localization of core and its colocalization with E2. Quantitative confocal immunofluorescence (IF) analysis of the sub-cellular localization of core and AP2M1 and core-E2 colocalization in Huh-7.5 cells. A. A representative merged image of endogenous AP2M1 (blue) and the LD marker, Bodipy (green), in naive Huh-7.5 cells. B–D. Merged images of Huh-7.5 cells infected with J6/JFH HCV stained for core (red), the LD marker, Bodipy (green), and AP2M1 (blue). E. Percent colocalization of the indicated signals in naive (white) or infected (black) cells by a quantitative colocalization analysis of A–D. F. A four channel merged image. The yellow arrows in the inset indicate colocalization of core and AP2M1 to LD. G–J. Representative merged images and quantitative colocalization analysis of AP2M1 (red) and the lipid marker, LipidTOX (blue), in Huh-7.5 cells transfected with plasmids expressing AP2M1-mCherry alone (G) or AP2M1-mCherry with WT core (H) or core Y136A mutant (I). Core expression (green) in the cells shown in panels H and I is demonstrated in the respective bottom panels. (K–P) Representative merged images and quantitative colocalization analysis of core (red) and: K. Bodipy (green), demonstrating increased localization of core to LD in Huh-7.5 cells electroporated with J6/JFH HCV RNA harboring the Y136A core mutation (right panel) compared with WT core (left panel). L. Bodipy (green) in control (NT) cells (left panel) or AP2M1 depleted (right panel) Huh-7.5 electroporated with J6/JFH HCV RNA, showing a dramatic localization of core to LD in AP2M1 depleted cells. M. TGN46 (green), demonstrating decreased localization of core to TGN in Huh-7.5 cells electroporated with J6/JFH RNA harboring the Y136A core mutation (right panel) compared with WT core (left panel). N. TGN46 (green) in control (NT) cells (left panel) or AP2M1 depleted (right panel) Huh-7.5 electroporated with J6/JFH HCV RNA, showing decreased localization of core to TGN in AP2M1 depleted cells. O. E2 (green), demonstrating decreased colocalization of core and E2 in Huh-7.5 cells electroporated with J6/JFH RNA harboring the Y136A core mutation (right panel) compared with WT core (left panel). P. E2 (green) in control (NT) cells (left panel) or AP2M1 depleted (right panel) Huh-7.5 electroporated with J6/JFH HCV RNA, showing decreased colocalization of core and E2 in AP2M1 depleted cells. Representative images at ×60 magnification are shown. Graphs represent quantitative colocalization analysis of Z stacks using Manders' coefficients. Values indicate mean M2 values represented as percent colocalization (the fraction of green intensity that coincides with red intensity or in the case of Figures G–I, the fraction of blue intensity that coincides with red intensity) ± s.d. (error bars); n = 10–15. * p<0.05, ** p<0.01, *** p<0.001.

To test our hypothesis that the defect in HCV assembly resulting from YXXΦ core mutations or AP2M1 silencing correlates with alterations in the sub-cellular localization of AP2M1 and/or core, we performed a quantitative confocal immunofluorescence (IF) analysis. 10–15 randomly chosen cells were analyzed for each category for the degree of localization of core or AP2M1 to various intracellular compartments using ImageJ (JACoP) software and Manders' Colocalization Coefficients (MCC). The latter were chosen, as they strictly measure co-occurrence independent of signal proportionality [77]. Endogenous AP2M1 minimally colocalized with the LD marker, Bodipy, in naive Huh-7.5 cells, with 8.2% of LD staining positive for AP2M1 (Figures 5A, 5E). In contrast, infection with J6/JFH virus appeared to significantly increase the localization of AP2M1 to LD, with 40±8% of LD being AP2M1 positive (Figures 5B, 5E) (p-value = 0.0006). Similarly, and as previously described [11], [22], [74], core was partially localized to LD (37±14% of core positive LD) (Figures 5C, 5E). Furthermore, the partial colocalization of core with AP2M1 occurred in part on LD (Figures 5D, 5F).

To test whether core is involved in mediating the increased localization of AP2M1 to LD measured in HCV infected cells, we overexpressed AP2M1-mCherry either alone or in combination with WT core or Y136A core mutant by transfecting Huh-7.5 cells. LD were labeled with HCS LipidTOX (Invitrogen). Similarly to naive cells, AP2M1 was minimally localized to LD in Huh-7.5 cells overexpressing AP2M1 alone (8±2% of LD positive for AP2M1) (Figures 5G, 5J). In contrast, as in infected cells, when co-expressed with WT core, AP2M1 appeared to significantly accumulate at LD, with 51.8±20% of LD being AP2M1 positive (p-value = 4.8×10^−5^) (Figures 5H, 5J). No such increase in colocalization was demonstrated, however, when AP2M1 was co-expressed with core harboring the Y136A mutation (with 10% AP2M1 positive LD) (p-value = 0.001) (Figures 5I, 5J), suggesting that core's YXXΦ motif may mediate recruitment of AP2M1 to LD.

We next tested the effect of disruption of the core-AP2M1 interaction on core localization to LD, ER, and TGN and its colocalization with the E2 envelope protein in cells electroporated with J6/JFH HCV RNA. As previously shown, core localized to all these intracellular compartments [11], [74] (with percent colocalization ranging from 30 to 45%). Interestingly, localization of core harboring the Y136A mutation to LD was significantly greater than that of WT core (78±13% vs. 32±16%, respectively, p-value = 2.7×10^−6^) (Figure 5K). Similarly, the percent colocalization of core with the LD marker was dramatically increased from 25±10% in NT cells to 87±6.6% following silencing of AP2M1 (p-value = 1.55×10^−8^) (Figure 5L). While neither the Y136A core mutation nor AP2M1 depletion had an apparent effect on core colocalization with the ER marker, calreticulin (Figure S5 in Text S1), both were associated with a significant decrease in core colocalization with the TGN marker, TGN46 (Figures 5M, 5N) and the E2 envelope protein (Figures 5O, 5P).

Together, these results suggest that core's interaction with AP2M1 facilitates recruitment of AP2M1 to LD and that the observed defect in HCV assembly following disruption of the core-AP2M1 interaction is associated with accumulation of core on LD, decreased core colocalization with E2, and impaired core trafficking to TGN.

### AAK1 and GAK regulate core-AP2M1 binding and are essential for HCV assembly

**Figure 6:**
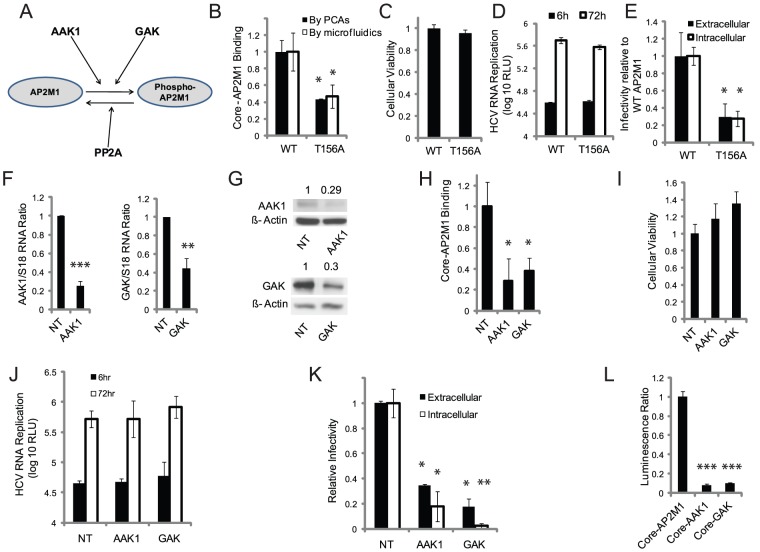
AAK1 and GAK regulate core-AP2M1 binding and are essential for HCV assembly. A. Regulatory mechanisms of AP2M1 binding to host cargo proteins harboring YXXΦ signals. B. Binding of core to wild type and T156A AP2M1 mutant by PCAs (black) and microfluidics (white). (C–E) Huh-7.5 were transfected with plasmids encoding WT or T156A AP2M1 mutant and electroporated with J6/JFH(p7-Rluc2A) 48 hr posttransfection. C. Cellular viability by alamarBlue-based assays at 48 hr posttransfection relative to WT AP2M1 control. D. HCV RNA replication in cells overexpressing WT or T156A AP2M1 mutant by luciferase assays at 6 hr (black) and 72 hr (white) postelectroporation with J6/JFH(p7-Rluc2A). E. Extracellular (black) and intracellular (white) infectivity by luciferase assays in naive Huh-7.5 cells infected with supernatants or cell lysates derived from the indicated cells, respectively, relative to WT control. (F–G) Huh7.5 cells were transfected with the corresponding siRNAs. F. Ratio of AAK1 (left) or GAK (right) to S18 RNA in these cells relative to NT sequences by qRT-PCR. G. Quantitative Western analysis. Numbers represent AAK1 (top) or GAK (bottom) to actin protein ratios relative to NT control. H. Core-AP2M1 binding by PCAs in Huh-7.5 cells depleted for AAK1 or GAK by siRNAs. Y axis represents luminescence ratio (the average luminescence signal detected in cells transfected with Gluc1-AP2M1 and Gluc2-core divided by the average of luminescence measured in NT cells transfected with Gluc1-AP2M1 and an empty Gluc2 vector with those transfected with Gluc2-core and an empty Gluc1 vector) relative to NT control. (I–K) AAK1 or GAK depleted cells were electroporated with J6/JFH(p7-Rluc2A). I. Cellular viability by alamarBlue-based assays in depleted cells relative to NT control. J. HCV RNA replication in these cells by luciferase assays at 6 hr (black) and 72 hr (white) postelectroporation. K. Extracellular (black) and intracellular (white) infectivity by luciferase assays in naive Huh-7.5 cells infected with supernatants or cell lysates derived from the indicated cells, respectively, relative to NT control. L. Core binding to AAK1 and GAK by PCAs. Y axis represents luminescence ratio relative to core-AP2M1 binding. Data represent means and s.d. (error bars) from at least two experiments in triplicates. RLU is relative light units. * p<0.05, ** p<0.01, *** p<0.001.

Phosphorylation of T156 within AP2M1 by the serine/threonine kinases AAK1 and GAK [51], [52], [53] stimulates binding of AP2M1 to cargo protein tyrosine signals and is transient due to dephosphorylation by PP2A [72] (Figure 6A). Indeed, calyculin A (a PP2A inhibitor) augmented the binding of core to AP2M1 (Figure 2D) and a T156A AP2M1 mutation impaired it (Figure 6B). To study the effect of overexpression of AP2M1 harboring the T156A mutation on infectious HCV production, Huh-7.5 cells were transfected with plasmids encoding either WT or AP2M1 T156A mutant. 48 hr posttransfection cells were electroporated with J6/JFH(p7-Rluc2A) HCV RNA and subjected to HCV RNA replication and infectivity assays, as described above. Overexpression of the T156A AP2M1 mutant had no effect on cellular viability and was dispensable for HCV RNA replication, yet significantly reduced extra-and intracellular infectivity compared with WT AP2M1 (Figures 6C–E), consistent with a dominant negative effect on HCV assembly.

To study our hypothesis that AAK1 and GAK are involved in regulating the interaction of AP2M1 with core, we conducted binding experiments in Huh-7.5 cells depleted for AAK1 or GAK by siRNAs (Figures 6F, 6G). Depletion of either AAK1 or GAK significantly decreased core-AP2M1 binding compared to NT, as measured by PCAs (Figure 6H), with no apparent cytotoxic effect (data not shown). AAK1 and GAK depleted cells were then electroporated with J6/JFH(p7-Rluc2A) HCV RNA. While depletion of either AAK1 or GAK had no cytotoxic effect and was dispensable for HCV RNA replication, it significantly reduced intracellular and extracellular infectivity (Figures 6I–K). The effect of AAK1 and GAK on HCV assembly did not result from their direct binding to core (Figure 6L). These results provide evidence for the involvement of AAK1 and GAK in the regulation of core-AP2M1 binding and in mediating HCV assembly. Moreover, they validate the importance of AP2M1 for efficient HCV assembly by a dominant-interfering approach, thus supporting our RNAi data.

### Pharmacological inhibitors of AAK1 and GAK disrupt core-AP2M1 binding and HCV assembly

**Figure 7:**
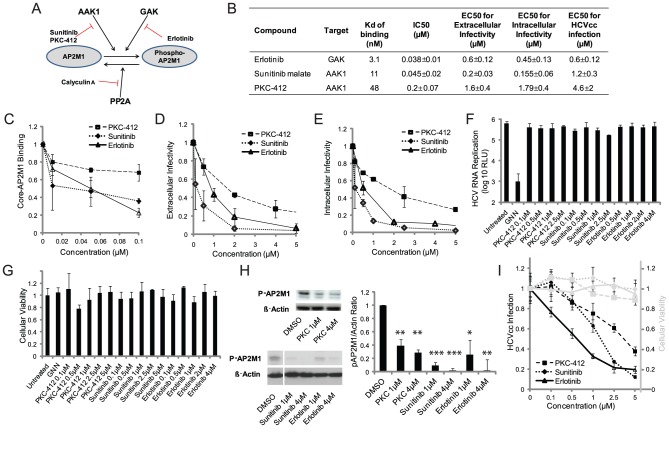
Pharmacological inhibition of core-AP2M1 binding and HCV assembly. A. AP2M1 regulators and the discovered inhibitors. B. The inhibitors' Kds of binding to AAK1 or GAK [78], IC50s for these compounds effect on core-AP2M1 binding, and EC50s for their effect on extracellular infectivity, intracellular infectivity, and viral infection with cell culture grown HCV (HCVcc). C. Inhibition of core-AP2M1 binding by the compounds measured by PCAs. D–G. Huh-7.5 cells electroporated with J6/JFH(p7-Rluc2A) were treated daily with either erlotinib, sunitinib or PKC-412 for 3 days. Supernatants and cell lysates were harvested at 72 hr and used to inoculate naive Huh-7.5 cells. Dose response curves of the inhibitors' effects on extracellular (D) and intracellular (E) infectivity relative to untreated controls. These compounds had no effect on HCV RNA replication (F) or cellular viability (G) by luciferase and AlamarBlue-based assays, respectively (GNN is a replication-defective polymerase mutant). H. The effect of the inhibitors on AP2M1 phosphorylation by Western analysis of cell lysates harvested following electroporation with J6/JFH(p7-Rluc2A) and treatment with the compounds in the presence of Calyculin A (Cal-A). Representative membranes blotted with anti-phopho-AP2M1 (p-AP2M1) and anti-actin antibodies, and quantitative analysis from 3 experiments are shown. Y axis represents pAP2M1/actin protein ratio relative to untreated controls. I. The inhibitors' effect on viral infection (black) and cellular viability (grey) in cells infected with HCVcc following 72 hr of daily treatment relative to untreated controls. Data represent means and s.d. (error bars) from at least three experiments in triplicates. RLU is relative light units. * p<0.05, ** p<0.01, *** p<0.001.

Analysis of heat maps and affinity assays of kinase inhibitors [78] revealed compounds, such as sunitinib and PKC-412, which bind AAK1, and erlotinib which binds GAK, with high affinities (nM range) [78] (Figures 7A, 7B). These compounds inhibited core-AP2M1 binding in a dose-dependent manner, as determined by PCAs (Figure 7C), with half maximal inhibitory concentrations (IC50s) of ∼0.04–0.2 µM. When used to treat Huh-7.5 cells electroporated with the J6/JFH(p7-Rluc2A) HCV genome, these compounds had a dramatic dose-dependent effect on extra- (Figure 7D) and intracellular infectivity (Figure 7E) at 72 hr (with half maximal effective concentration (EC50s) of 0.15–1.8 µM) (Figure 7B and Text S1). There was no effect on HCV RNA replication and no apparent cellular toxicity (Figures 7F, 7G). Indeed, the inhibitory effect on core-AP2M1 binding and infectivity was associated with reductions in phospho-AP2M1 levels in the relevant cells by Western analysis (Figure 7H, Text S1). Last, these compounds significantly inhibited viral infection in Huh-7.5 cells infected with tissue culture grown HCV (titer: 6.3×10^5^ TCID_50_/ml) (Figures 7I, 7B, Text S1). These results provide pharmacological validation for the involvement of AAK1 and GAK in regulating the core-AP2M1 interaction and for AP2M1's role in HCV assembly. Furthermore, they provide candidate compounds targeting assembly.

## Discussion

We combined novel proteomic technologies with molecular virology, genetic, RNAi, dominant interfering, and pharmacological approaches, to validate an interaction between a heretofore unrecognized tyrosine motif within HCV core and AP2M1 that is essential for viral assembly, and to identify inhibitors that disrupt this interaction and inhibit HCV assembly.

Our results suggest that core's AP2M1 binding motif is required for HCV assembly *in vitro*. Y136A and V(Φ)139A core YXXΦ mutations significantly impaired AP2M1 binding, in line with the key role played by these residues in facilitating recognition of cargo proteins by AP2M1 [47], [48], [49]. Furthermore, these core mutations dramatically impaired HCV assembly, in correlation with their effect on AP2M1 binding. These results are consistent with prior alanine scanning and deletion analysis demonstrating a significant effect of either quadruple alanine substitutions or a larger deletion harboring the YXXΦ core residues on infectious virus production [73], [79]. Emergence of primary-site revertants that coincided with phenotypic reversion of the infectivity phenotype, as we detected, further indicates the requirement of maintaining a functional YXXΦ motif for supporting HCV replication *in vitro*. This motif is highly conserved across all natural HCV isolates available in databases to date, suggesting that there is a requirement for the AP2M1 binding residues for productive viral infection *in vivo*. The functional interchangeability of core's YXXΦ motif with homologous motifs suggests that it exerts its function via interactions with host cell proteins. Indeed, stable and transient functional RNAi approaches as well as dominant interfering and pharmacological approaches consistently demonstrated that AP2M1 is essential for efficient HCV assembly. Core's YXXΦ motif thus represents the first signal mediating binding of clathrin APs discovered and validated in the Flaviviridae family.

Binding of core to AP2M1 provides a mechanism by which HCV hijacks endocytic functions for its assembly. Few other viruses, such as HIV [57], [58], EIAV [54], [55], [56], and HBV [80], [81], [82] have been shown to rely on early endocytic functions for their assembly and budding. Unlike the established role of late endocytic functions in facilitating membrane fission events at the site of envelopment (reviewed in [83]), the role mediated by these early endocytic proteins in the late stages of viral life cycles is, however, less studied.

While most studies thus far have focused on AP2M1's role in mediating endocytosis of CCV from the plasma membrane to endosomes, there is evidence that it may be involved in mediating additional intracellular membrane traffic processes. AP-2 was shown to initiate assembly of clathrin coats on lysosomes [50]. Moreover, recent proteomic studies have revealed its presence on ER and Golgi membranes [84]. Contacts between endosomes and ER membranes [85] may help facilitate such recruitment of AP2M1 to the ER membrane. Interestingly, several endosomal membrane trafficking proteins have been previously shown to be recruited to LD in naive cells [86], illustrating the dynamic nature of these intracellular compartments. Our study did not demonstrate significant localization of endogenous or exogenous AP2M1 to LD in either naive cells or cells overexpressing AP2M1 alone, respectively. Nevertheless, in the setting of overexpression of core or HCV infection, AP2M1 appeared to significantly localize to the surface of LD. Importantly, no such colocalization was demonstrated when AP2M1 was co-expressed with core harboring the Y136A mutation, suggesting that core's YXXΦ motif hijacks AP2M1 and facilitates its recruitment to LD for mediating HCV assembly.

Core has been previously shown to localize to various compartments including LD, ER, and TGN [22]. Our quantitative confocal microscopy experiments indeed demonstrated core localization to all these compartments. Nevertheless, increased localization of core to LD and decreased localization to TGN were demonstrated in cells electroporated with HCV RNA harboring a Y136A core mutation or depleted for AP2M1. These results indicate that the observed defects in infectious HCV production following disruption of the core-AP2M1 interaction are associated with altered sub-cellular localization of core. AP2M1, like other clathrin adaptors, is known to sort cargo proteins and mediate intracellular traffic (reviewed in [49]). It was previously shown that AP subunits are also involved in mediating intracellular trafficking of structural retroviral proteins. An interaction between Gag's YXXΦ motif with AP2M1 was shown to confine HIV-1 exit to distinct microdomains [58]. Similarly, an interaction between Gag and the δ subunit of the AP-3 complex has been shown to mediate trafficking of Gag to CD63-positive intracellular compartments and to be essential for HIV assembly [87]. Binding of AP2M1 to core's YXXΦ motif may thus offer a mechanism to facilitate intracellular trafficking of core. Disruption of core's interaction with DGAT1 is associated with accumulation of core on ER membranes and failure of core to traffic to LD, consistent with a defect in an early assembly stage [33]. Unlike the core-DGAT1 interaction, our data suggest that the rate limiting stage affected by disruption of the core-AP2M1 binding is transport of core away from LD. These data, combined with the reduction of core colocalization with E2 demonstrated in the context of the Y136A core mutation or AP2M1 depletion, suggest that core-AP2M1 binding may mediate a later stage of HCV assembly following accumulation of core on LD but prior to envelopment at the ER. Interestingly, we have recently identified conserved YXXΦ and dileucine motifs within the cytoplasmic tail of the E1 protein (data not shown). It is therefore tempting to speculate that core and E1 may interact on the same adaptor protein complex and that by binding both core and E1, AP2M1 may help facilitate trafficking of viral particles from their assembly sites on LD (where core is accumulated) to the envelopment sites at the ER (where the E1 protein is retained). Interestingly, such a mechanism has previously been described in HBV, where the envelope and capsid proteins both interact with γ2 adaptin as a means to traffic viral particles from assembly to budding sites [81]. Consistent with its role in mediating HCV assembly, disruption of core-AP2M1 binding reduced core localization to TGN, the compartment in which viral particles are thought to mature during egress. Since ER is not only the site of presumed viral particles envelopment but also the site of core synthesis and processing, it is not surprising that no apparent decrease in localization of core to ER was demonstrated as a result of either core mutation or AP2M1 depletion. We plan to determine whether E1 binds AP2M1 and whether this interaction is cooperatively involved in mediating assembly. Future studies using live cell imaging of core trafficking will help determine whether the core-AP2M1 interaction directly mediates core transport out of LD to the envelopment sites.

Late endocytic functions have been shown to be involved in mediating production of infectious HCV virus. Either expression of dominant negative forms of Endosomal Sorting Complexes Required for Transport (ESCRT) components [59] or their depletion reduced production of infectious virus [36], [60]. Moreover, inhibitors of endosomal motility disrupt HCV assembly and/or budding [61]. Nevertheless, AP-made vesicles bud into the cytoplasm, which is the topological reverse of HCV budding into the lumen of intracellular compartments. Based on this topology and our IF confocal microscopy analysis, it seems lower likely that AP2M1 itself promotes vesicle formation into the ER and the pinching-off reaction required for viral envelopment.

Functionally relevant interactions between YXXΦ motifs within structural viral proteins and AP2M1 have been previously described in retroviruses [54], [55], [56], [57], [58]. To the best of our knowledge, such interactions have not been previously reported in HCV or any other viral family except Retroviridae. Furthermore, AAK1 and GAK have not been reported to mediate a viral infection to date. Importantly, AP2M1's interactions with retroviral or host proteins have not been targeted pharmacologically. Using RNAi and pharmacological approaches, we show that AAK1 and GAK are involved in regulating core-AP2M1 binding. The role of AAK1 and GAK in stimulating binding of host cargo proteins to AP2M1, suggests that the HCV core protein may function as a cargo protein. Our data also suggest that AAK1 and GAK are host factors essential in mediating HCV assembly. Moreover, we discovered kinase inhibitors known to target AAK1 or GAK, which inhibit AP2M1 phosphorylation, disrupt binding of core to AP2M1, and inhibit HCV assembly as well as infectious virus production. While the discovered compounds are not entirely selective (like the majority of compounds [88]), there is evidence to support their relatively good selectivity. Eventhough AAK1 and GAK are not among the cancer targets of these compounds, they are very potently bound and inhibited by sunitinib or PKC-412 and erlotinib, respectively (with Kds of binding that are lower or comparable to the major cancer targets). For example, the Kd of binding of erlotinib to its primary cancer target, EGFR, is ∼1 nM compared with a Kd of binding of 3.1 nM to GAK (SuperNova Life Science 2008, Human Kinome Heat Map, http://www.supernovalifescience.com/HM/HM%2041.pdf and [78]). Importantly, the Kds of binding of erlotinib to all other studied host protein kinases are significantly higher (with most being >1,000–10,000 nM). Moreover, these compounds had no effect on HCV RNA replication, yet significantly inhibited assembly. Last, their effect on core-AP2M1 binding and their overall effect on HCV replication (Figure 7I) correlated with their binding affinity to AAK1 or GAK. The IC50 values of the compounds for core-AP2M1 binding were 0.04–0.2 µM, whereas their EC50 values for production of infectious virus in cells were 0.155–1.8 µM. Such ∼10–25-fold differences between IC50s and EC50s values are typical for kinase inhibitors [89]. It is possible that a limited bioavailability (determined by the rates of entry to the cells and/or active pumping out of the cells) of the compounds accounted for these differences [89]. We hypothesize that improved drug delivery and optimization of the compounds after structure-activity relationship analysis will improve their potency as antiviral agents.

Interestingly, erlotinib has been recently reported to inhibit HCV endocytosis via its effect on EGFR [90]. While sunitinib and PKC-412 do not target EGFR, as described above the latter represents the only other known major target of erlotinib besides GAK. Inhibition of GAK-mediated assembly thus represents another mechanism of action of erlotinib in the HCV life cycle, besides its effect on EGFR-mediated entry. Our data suggest that both mechanisms accounted for the effect of erlotinib on HCV infection in cells infected with culture grown HCV (Figure 7I). Nevertheless, its dramatic effect on infectious virus production, measured by the infectivity assays in cells electroporated with HCV RNA (Figures 7D, 7E), confirms its independent effect on viral assembly. Importantly, erlotinib has already demonstrated an antiviral activity *in vivo*, by effectively eradicating HCV infection in humanized mice infected with HCV, without inducing any apparent toxicity [90]. Moreover, a recent case report describes rapid clearance of HCV RNA in serum of a patient undergoing treatment with erlotinib for recurrent hepatocellular carcinoma [91]. While attributed to its effect on EGFR, our data suggest that the observed antiviral effect likely resulted from its dual effects on EGFR and GAK. Erlotinib, sunitinib, and PKC-412 may represent a pharmacological research tool not only for studying host cell mechanisms involving AAK1 or GAK but also for studying interactions of AP2M1 with cargo proteins. Furthermore, these compounds may serve as an attractive strategy for treatment of HCV. Such a host-centered approach may potentially be associated with a higher genetic barrier for resistance and may provide an efficient means to combat all HCV genotypes, unlike genotypic specificity associated with some drugs that target viral enzymatic functions [92]. Erlotinib and sunitinib are already approved anti-cancer drugs and PKC-412 is at an advanced clinical development stage. Our studies revealed no effect of these compounds on cellular viability at the concentrations used. Similarly to drugs used in current standard of care anti-HCV regimens, side effects, including some that are significant, have been reported in patients receiving erlotinib, sunitinib or PKC-412 for the treatment of various malignancies. Nevertheless, overall they seem to be well tolerated over prolonged treatment courses [93], [94], [95], [96], [97]. Importantly, these drugs are delivered orally, thus avoiding the inconvenience and complications related to parenteral administration. We hypothesize that more selective and potent inhibitors of AAK1, GAK and/or core-AP2M1 binding can be obtained. Nevertheless, because erlotinib, sunitinib, and PKC-412 have already been extensively used in humans (albeit for a different indication) there may be an opportunity for repurposing them as antivirals. As recently discussed by Jilg N and Chung RT in respect to erlotinib [98], these compounds may find immediate use as components of next generation anti-HCV strategies with particular utility among patients failing to respond to standard of care regimens.

AP2M1 represents an example for an overlapping host mechanism required by HCV and lentiviruses. Indeed, a common evolutionary origin of these seemingly unrelated viruses has been previously proposed [99]. We now plan to study the role of AAK1 and GAK in mediating infectious virus production of other viruses that hijack AP2M1, such as HIV. Conservative requirement for AAK1 and GAK across viral families may suggest that the identified inhibitors could potentially be used for treating HCV-HIV co-infection and possibly a broader spectrum of viral infections.

Finally, our results demonstrate the utility and advantages of the microfluidics and PCAs approaches for studying viral-host P-PIs. These sensitive formats allow studying weak and transient interactions. Protein synthesis by mammalian lysates in the presence of microsomal membranes provides natural conditions required for protein folding, thus facilitating studies of membranous proteins by the microfluidics approach. The PCAs format allows detection of P-PIs involving membrane proteins in the relevant cell model and appropriate sub-cellular compartments [68], [100] (unlike most yeast two-hybrid systems where the interaction occurs in the nucleus). By allowing nanoliter protein consumption, the microfluidics technology eliminates the need for high level protein expression and purification, which hinders the design of other protein arrays. Their scalability and ability to study pharmacological inhibition of interactions, makes these platforms ideal for high-throughput screening not only for P-PIs but also for inhibitors of identified P-PIs (as we have previously done for protein-RNA interactions [66]).

Taken together, these results have exciting implications with respect to mechanisms of HCV assembly and design of novel antiviral strategies.

## Materials and Methods

### Plasmids

ORFs encoding AP2M1, GAK, AAK1, TFR, SPIRE, RAC1, ARPC, and NESI were picked from the Human ORFeome library of cDNA clones [101] (Open biosystems) and recombined into either pcDNA-Dest40 (for C-terminal V5-his tagging), pGLuc (for *Gaussia Princeps* luciferase fragment (Gluc) tagging) [68], and/or pCherry (for mCherry fluorescence protein tagging) vectors using gateway technology (Invitrogen). ORFs encoding T7-tagged full length core and NS3 were amplified from described vectors [102] and ligated into pcDNA3.1 (Invitrogen). pFL-J6/JFH(p7-Rluc2A) [73] was a gift from Dr. C.M. Rice [103]. The YXXΦ core mutations and AP2M1 mutations were introduced into these plasmids by site-directed mutagenesis (using the QuikChange kit (Stratagene)).

### Antibodies and compounds

See Tables S1 and S2 in Text S1.

### RNAi and rescue of gene silencing

40–100 nM individual or pooled siRNAs were transfected into cells using silMPORTER (Upstate, Millipore) 48 hr prior to HCV RNA electroporation. 5 individual MISSION Lentiviral Transduction Particles (Sigma) harboring short hairpin RNAs (shRNAs) targeting various sites in the AP2M1 RNA and a control shRNA were used to transduce Huh-7.5 cells according to the manufacturer's protocol. RNAi reagents used in this study are summarized in Table S3 in Text S1. AP2M1 rescue was performed by transduction of Huh-7.5 cells stably depleted for AP2M1 with lentiviruses expressing shRNA-resistant AP2M1 48 hr prior to electroporation with HCV genome.

### Microfluidics affinity assays

Device fabrication and design were done essentially as described [65]. V5-his-tagged human proteins and T7-tagged viral proteins were expressed off the chip by mammalian *in vitro* transcription/translation mixture (TNT) (Promega) (in the presence of microsomal membranes). The device was subjected to surface patterning, resulting in a circular area coated with biotinylated anti-his antibodies within each unit cell [66], [67]. Protein-protein binding experiments were performed as described [67]. Briefly, human bait proteins were loaded into the device and bound to the surface anti-his antibodies. Viral and human proteins were incubated in the chip and labeled with anti-T7-Cy3 and anti-V5-FITC antibodies, respectively. Interactions were trapped mechanically by MITOMI [65], [66], [67]. After a brief wash to remove untrapped unbound material, the trapped protein complexes were detected by an array scanner (Tecan). The ratio of bound viral prey to expressed human bait protein was calculated for each data point by measuring the ratio of median signal of Cy3 to median signal of FITC. Protein concentration in lysates were determined by quantitative Western analysis against standard curves of T7-tagged proteins. Experiments were conducted at least three times, each time with >20 replicates. See Figure S1 in Text S1 for a detailed protocol.

### Cell cultures

Huh-7.5 cells and 293 T cells were maintained in Dulbecco's modified minimal essential medium (Gibco) supplemented with 1% L-glutamine (Gibco), 1% penicillin, 1% streptomycin (Gibco), 1X nonessential amino acids (Gibco), and 10% fetal bovine serum (Omega Scientific). Cell lines were passaged three times a week after treatment with 0.05% trypsin–0.02% EDTA and seeding at a dilution of 1∶4.

### Protein-fragment complementation assays

Binding assays were performed essentially as described [68]. Combinations of plasmids encoding prey (A) and bait (B) proteins, each fused to a fragment of the *Gaussia Princeps* luciferase protein (Gluc1 and Gluc2) or control vectors were cotransfected into 293 T or Huh-7.5 cells plated in 96-well plates in triplicates. At 24 hr posttransfection, cells were lysed and subjected to standard luciferase reporter gene assays using *Renilla* luciferase assays system (Promega). Results were expressed either as luminescence or luminescence ratio. The latter represents the average luminescence signal detected in cells transfected with Gluc1-A and Gluc2-B divided by the average of luminescence measured in control wells transfected with Gluc1-A and an empty Gluc2 vector with those transfected with Gluc2-B and an empty Gluc1 vector. Competition assays and studies designed to determine inhibitory effect of compounds or siRNAs were performed as above, except that binding was measured in the presence of excess free proteins, the inhibitors, or siRNAs, respectively. Experiments were conducted at least three times in triplicates.

### Co-immunoprecipitations

Membranes were prepared from ∼20×10^6^ Huh-7.5 cells infected with HCV (J6/JFH) [6], as we have previously described [104]. Briefly, cells were collected by trypsinization, washed once with PBS and resuspended in HME buffer (20 mM HEPES [pH 7.4], 1 mM EDTA, 2 mM MgCl2), supplemented with phenylmethylsulfonyl fluoride to a final concentration of 1 mM and a protease inhibitors cocktail (Sigma). Cells were lysed by two cycles of freeze-thaw in dry ice-ethanol and then passaged through a 27.5-gauge needle 10 times. Nuclei were removed by centrifugation at 250×g for 10 min, and the postnuclear supernatant was subjected to ultracentrifugation at 100,000×g for 30 min to obtain the membrane preparation. All steps were done at 4°C. Total membrane proteins were diluted in 1 ml TDB buffer (2.5% Triton X-100, 25 mM triethanolamine-Cl [pH 8.6], 20 mM NaCl, 5 mM EDTA, 0.2% NaN3) [104] and incubated for 30 min at 37°C with 100 nM calyculin-A or DMSO. Due to the weak and transient nature of the interactions, 25 mM dithiobis-succinimidyl-propionate (DSP) cross-linker (Pierce) was added to allow covalent binding of the already bound interacting proteins. Samples were incubated for 2 hr on ice. 1 M Tris was added to stop the DSP activity. Lysates were then clarified by 10 min centrifugation at 1000×g, followed by 30 min ultracentrifugation of the supernatants at 100,000×g. Membrane pellets were resuspended in 100 µl HME buffer (20 mM NaHEpes (PH 7.4), 1 mM EDTA (Ph 8), 2 mM MgCl2), and TDB buffer was added for a final volume of 1 ml. Samples were incubated overnight with either anti-AP2M1 antibodies, anti-core antibodies or IgG controls, and protein G magnetic beads (Millipore). Immunoprecipitates were analyzed by Western blotting. Experiments were conducted twice in duplicates.

### *In vitro* transcription of viral RNA and transfection

HCV RNA was generated and delivered into Huh-7.5 cells, as previously described [6], [73]. Briefly, RNA was synthesized from XbaI linearized J6/JFH(p7-Rluc2A) template using the T7 MEGAscript kit according to the manufacturer's protocol (Ambion). Reaction mixtures were incubated for 3 hr at 37°C and then subjected to DNase treatment for 15 min at 37°C. Viral RNA was purified using the RNeasy kit (Qiagen). RNA was quantified by absorbance at 260 nm, and its quality was verified by agarose gel electrophoresis. Subconfluent Huh-7.5 cells were trypsinized and collected by centrifugation at 700 g for 5 min. The cells were then washed three times in ice-cold RNase-free PBS (BioWhittaker) and resuspended at 1.5*10^7^ cells/ml in PBS. 5 µg of the *in vitro* transcribed wild type or J6/JFH(p7-Rluc2A) mutant RNA was mixed with 400 µl of washed Huh-7.5 cells in a 2 mm-gap cuvette (BTX) and immediately pulsed (0.82 kV, five 99 µs pulses) with a BTX-830 electroporator. After a 15 min recovery at 25°C, cells were diluted in 30 ml of prewarmed growth medium and plated into 96, 24, 6-well and P100 tissue culture plates.

### HCV RNA replication by luciferase assays

HCV RNA replication was measured at 6–9 hr, and 72 hr postelectroporation, as described [73]. Electroporated cells plated in quadruplicates in 96-well plates were washed twice with PBS and lysed with 30 µl of *Renilla* lysis buffer (Promega). Following 15 min of shaking at RT, luciferase activity was quantified using a *Renilla* luciferase substrate (Promega) and a Tecan luminometer (Tecan) according to the manufacturers' protocols.

### Extracellular infectivity

Culture supernatants of Huh-7.5 cells electroporated with J6/JFH(p7-Rluc2A) RNA and plated in P100 dishes were harvested at 72 hr postelectroporation, clarified (with a 0.22-µm-pore size filter) and used to infect naïve Huh-7.5 cells for 72 hr in triplicates before lysis in *Renilla* lysis buffer (Promega). Luciferase activity in 20 µl of cell lysates was quantified as described above. To determine the effect of erlotinib, sunitinib, and PKC-412 on infectious virus production, electroporated cells were grown in the presence of the inhibitors with daily medium changes for 72 hr prior to collection of supernatants or cell lysates. Results represent log10 RLU values per 10 cm tissue culture dish. Experiments were repeated three times, each time with quadruplicates.

### Intracellular infectivity assays

As described [73], 72 hr postelectroporation with J6/JFH(p7-Rluc2A) RNA cells were trypsinized, collected by centrifugation, resuspended in 500 µl medium, lysed by freeze-thaw cycles, and pelleted twice at 3,650×g. Clarified supernatants diluted in complete medium were used to inoculate naive Huh-7.5 cells in triplicates, followed by lysis and luciferase assays at 72 hr. Results represent log10 RLU values per 10 cm tissue culture dish. Experiments were repeated three times, each time with quadruplicates.

### Virus titration

Extracellular and Intracellular titers were determined by limiting dilution assays based on immunohistochemical staining for core. 50% tissue culture infectious dose (TCID_50_) was calculated, as described [6]. Results are expressed as TCID_50_/ml. Minimal titers measured with the ΔE1–E2 mutant were used for background subtraction.

### Core ELISA

The concentration of released core protein was measured in clarified cell culture supernatants harvested at 72 hr postelectroporation by ELISA (Cell Biolabs) against standard curves of recombinant core antigen, according to the manufacturer's instructions.

### Viability assays

Following 24, 48, and 72 hrs of treatment with inhibitory compounds or silencing with siRNAs, cells were incubated for 2–4 hrs at 37°C in the presence of 10% AlamarBlue reagent (TREK Diagnostic Systems), as described [66]. Plates were then scanned and fluorescence was detected by using FLEXstation II 384 (Molecular Devices, Inc.).

### Infection studies

6×10^3^ Huh-7.5 cells seeded in 96-well plates were infected in triplicates with cell culture-grown HCV J6/JFH (titer: 1.2×10^5^ TCID_50_/ml). Extracellular and intracellular infectivity were measured by focus formation assays [6] in naive Huh-7.5 cells infected with supernatants or clarified cell lysates derived from the infected cells at 72 hr postinfection.

To determine the antiviral effect of erlotinib, sunitinib, and PKC-412, 6×10^3^ Huh-7.5 cells seeded in 96-well plates were infected in triplicates with cell culture-grown HCV (J6/JFH(p7-Rluc2A)) (titer: 6.3×10^5^ TCID_50_/ml) with an MOI (multiplicity of infection) of 0.1.

6 hr after infection and daily thereafter, cells were washed and medium was replaced with medium containing serial dilutions of the inhibitory compounds. At 72 hr, samples were subjected to viability assays, followed by standard luciferase assays (Promega).

### Analysis of revertants

Huh-7.5 cells electroporated with J6/JFH(p7-Rluc2A) harboring the Y136A or V139A mutations were propagated. Culture supernatants were harvested every few days and used to inoculate naive Huh-7.5 cells for determination of extracellular infectivity by luciferase assays 72 hr following inoculation. Total cellular RNA was extracted from clones demonstrating reversion of the infectivity phenotypone (at day 8 postelectroporation for Y136A clones and day 14 for V139A mutant clones) using TRIzol reagent (Invitrogen). Reverse transcription reaction and PCR amplification were performed using Superscript One-Step reverse transcriptase PCR (RT-PCR) kit (Invitrogen). A ∼1-kb segment harboring the core coding sequence was amplified. The PCR products were purified from agarose gels by Ultra Clean 15 DNA purification kit (MoBio) and subjected to automatic sequencing on an ABI Prism 377 DNA sequencer (Sequetech). Presence of primary site reversion was confirmed by two independent sequences using forward and reverse oligos.

### RNA extraction and qRT-PCR

Total RNA was isolated from cells or 1 ml cell culture supernatants using TRIzol (Invitrogen) or QiaAmp UltraSens kit (Qiagen), respectively. qRT-PCRs mixtures were assembled in triplicates using 0.5 µg or 4 µl RNA and High-Capacity RNA-to-cDNA (Applied Biosystems). TaqMan reagents are listed in Table S4 in Text S1. Amplification and analysis were performed using StepOnePlus Real-Time-PCR system (Applied Biosystems). S18 was used as a control.

### Western blot

Cell lysates were subjected to Western analyses using primary antibodies followed by HRP-conjugated secondary antibodies. Bands intensity was quantified using NIH Image.

### Quantitative immunofluorescence confocal microscopy

Huh-7.5 cells were electroporated with J6/JFH HCV RNA or infected with culture grown J6/JFH virus, seeded onto coverslips, and incubated for 72 hr at 37°C. Cells were fixed with 4% paraformaldehyde in PBS, washed with PBS, permeabilized with 0.1% Triton X-100 in PBS for 5 min, and blocked for 1 hr in PBS containing 1% BSA. Fixed cells were incubated with primary antibodies against either the core protein, AP2M1, TGN46, calreticulin or E2 at room temperature for 1 hr (see Table S1 in Text S1). Secondary antibodies (goat anti-rabbit Alexa Fluor 488, goat anti-mouse Alexa Fluor 594, chicken anti-goat IgG Alexa Fluor 647, and goat anti-human Alexa Fluor 647) (Invitrogen) were incubated for 1 hr at room temperature. LD staining with Bodipy-488/503 (Invitrogen) was performed as described [74]. Cover slips were then washed three times in PBS, and mounted with ProLong Gold antifade reagent (Invitrogen). Alternatively, Huh-7.5 cells were co-transfected with plasmids expressing AP2M1-YFP and/or Core-mCherry. LD were stained with HCS LipidTOX (Invitrogen). All slides were analyzed using Zeiss LSM 510 confocal microscope. Colocalization was quantified in 10–15 randomly chosen cells from each sample using ImageJ (JACoP) colocalization Software and Manders' Colocalization Coefficients (MCC). Threshold values were determined using auto_threshold (plugin, ImageJ). Only pixels whose red and green intensity values were both above their respective thresholds were considered to be pixels with colocalized probes. MCCs were then calculated as the fractions of total fluorescence in the region of interest that occurs in these “colocal” pixels (with a higher value representing more colocalization). M2 coefficient values represented as mean percent colocalization are shown.

### Statistical analysis

IC50s and EC50s were measured by fitting data to a three parameter logistic curve, as described [66]. P-values were calculated using one-tailed unpaired Student's t test.

## Supporting Information

Text S1 **The Supporting Information file includes Supporting Results, Supporting Methods, Figures S1, S2, S3, S4, S5, Tables S1, S2, S3, S4, Supporting References.**
(PDF)
