# Supplementary material for: Identification and Targeting of an Interaction between a Tyrosine Motif within Hepatitis C Virus Core Protein and AP2M1 Essential for Viral Assembly
Source: PLoS Pathog. 2012 Aug 16;8(8):e1002845. doi: 10.1371/journal.ppat.1002845 (PMC3420927; doi:10.1371/journal.ppat.1002845)
Supplement: Text S1 — The Supporting Information file includes Supporting Results, Supporting Methods, Figures S1, S2, S3, S4, S5, Tables S1, S2, S3, S4, Supporting References. (PDF) [file ppat.1002845.s001.pdf]

**Text S1: Supporting Information**

# Identification and targeting of an interaction between a tyrosine motif within hepatitis C virus core protein and AP2M1 essential for viral assembly

Gregory Neveu[1*], Rina Barouch-Bentov[1*], Amotz Ziv-Av[1], Doron Gerber[2], Yves Jacob[3], and Shirit Einav[1]

[1]Department of Medicine, Division of Infectious Diseases and Geographic Medicine, and Department of Microbiology and Immunology, Stanford University School of Medicine, Stanford, California.
[2]The Mina & Everard Goodman Faculty of Life Sciences and The Nanotechnology Institute, Bar-Ilan University, Ramat-Gan, 52900, Israel.
[3]Department of Virology, Unité de Génétique, Papillomavirus et Cancer Humain (GPCH), Pasteur Institute, Paris, 75724, France

**This file includes**

Supporting Results
Supporting Methods
Figures S1-5
Tables S1-4
Supporting References

## Supporting Results

**The effect of erlotinib, sunitinib, and PKC-412 on AP2M1 phosphorylation.** To determine the effect of the inhibitory compounds on AP2M1 phosphorylation, Huh-7.5 cells harboring J6/JFH(p7-Rluc2A) RNA were treated daily with various concentrations of the compounds or with DMSO. Since AP2M1 phosphorylation is transient (due to the activity of the phosphatase PP2A[1]), to allow capturing of the phosphorylated AP2M1 state, lysates were prepared at 72hr following a 30 min incubation of the cells with 100nM of the PP2A inhibitor, calyculin A (Cal-A) or a DMSO control. Samples were then subjected to SDS-PAGE and blotting with antibodies targeting either the phosphorylated AP2M1 form or actin. Indeed, significantly lower ratios of phosphorylated-AP2M1 to actin were measured in lysates prepared from cells treated with either sunitinib, erlotinib or PKC-412, compared with the DMSO control (Fig. 6H). Phospho-AP2M1 to actin ratios were progressively decreased by increasing concentrations of sunitinib and erlotinib in a dose-dependent manner. These results suggest that AAK1 or GAK are potently inhibited by these compounds in Huh-7.5 cells harboring infectious HCV.

## Supporting Methods

**The effect of the compounds on HCV RNA replication, intracellular infectivity, extracellular infectivity, and HCV replication.**

To determine the effect of erlotinib, sunitinib, and PKC-412 on infectious virus production, Huh-7.5 cells were electroporated with J6/JFH(p7-Rluc2A) HCV genome [2], plated in 6-well plates, and treated daily with serial dilutions of the compounds or DMSO. At 72 hr postelectroporation, cellular viability was measured by alamarBlue-based assays followed by luciferase assays for determination of HCV RNA replication. Cell culture supernatants and lysates were collected at 72 hr from parallel samples and used to inoculate naive Huh-7.5 cells for determination of extracellular and intracellular infectivity, respectively. Luciferase assays were performed in these cells at 72 hr post inoculation.

To determine the effect of these compounds on HCV replication, $6x10^3$ Huh-7.5 cells seeded in 96-well plates were infected in triplicates with cell culture-grown HCV (J6/JFH(p7-Rluc2A)) tittered at $6.3x10^5$ $TCID_{50}$/ml with an MOI (multiplicity of infection) of 0.1. At 6 hr postelectroporation, cells were washed and medium was replaced with serial dilutions of the inhibitory compounds. Cells were treated daily for 72 hr and then subjected to viability assays followed by standard luciferase assays.

Alternatively, $6x10^3$ Huh-7.5 cells seeded in 96-well plates were infected in triplicates with cell culture-grown HCV J6/JFH (titer: $1.2x10^5$ $TCID_{50}$/ml with an MOI of 0.1, and subjected to focus formation assays, as described [3]. Following 72 hr of daily treatment with the compounds, cells were fixed in 4% formaldehyde and permeabilized with saponin. HCV core protein was detected with primary anti-core monoclonal and secondary goat anti-mouse Alexa 594-conjugated antibodies. Foci were counted under an inverted microscope.

Experiments were repeated twice, each with 4 replicates. Signal was normalized to samples grown in the presence of the corresponding concentration of DMSO. EC50s were measured by fitting data to a three parameter logistic curve using the formula Y=a+(b-a)/(1+10^(X-c)) (a, b and c represent minimum binding, maximum binding and logEC50, respectively)(BioDataFit, Chang Bioscience, Inc).

## Supporting Figures

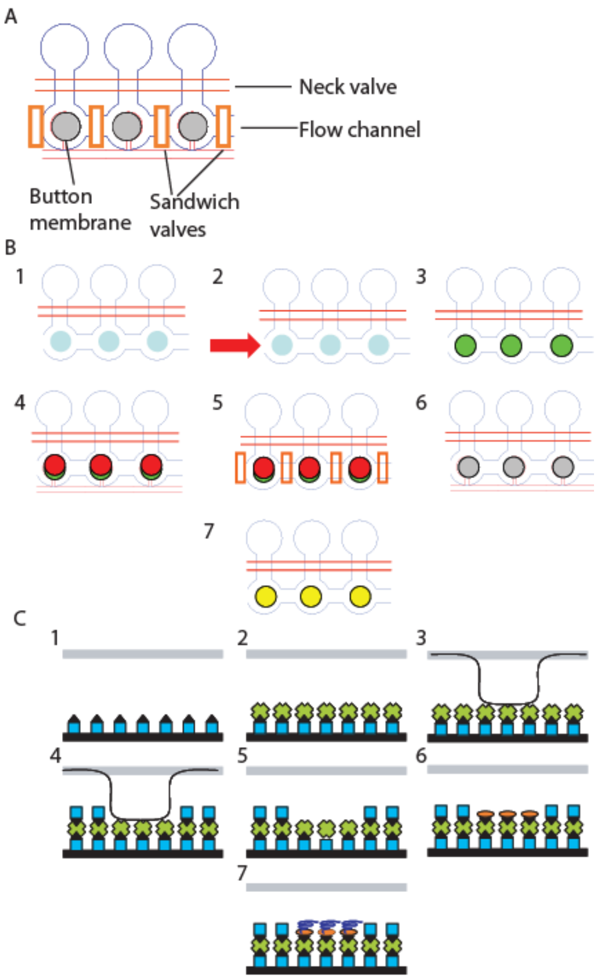


**Figure S1. Microfluidic-based protein-protein binding assay.**

3 individual unit cells (out of hundreds in a microfluidic device) are shown in this scheme.

A. Compartments and micromechanical valves. A valve is created where a control channel crosses a flow channel. The resulting thin membrane in the junction between the two channels can be deflected by hydraulic actuation. Using multiplexed valve systems allows a large number of elastomeric microvalves to perform complex fluidic manipulations within these devices.

B. Experimental protocol. ● represents surface bound biotinylated anti-histidine antibodies. ● represents surface bound FITC-labeled bait human protein. ● represents Cy3-labeled prey viral protein.

1) The microfluidic device was bonded to a glass slide and subjected to surface patterning that resulted in a circular area coated with biotinylated anti-histidine antibodies within each unit cell (see c). 2) V5-his-tagged bait human proteins were expressed off the chip using *in vitro* transcription/ translation (TNT) mixture and were loaded into the device. 3) These proteins bound to the surface anti-his antibodies. 4) T7-tagged viral proteins were expressed off the chip by the same mammalian *in vitro* TNT mixture in the presence of microsomal membranes and loaded into the device along with FITC-labeled anti-V5 and Cy3-labeled anti-T7 antibodies. 5) The "sandwich valves" were closed to allow incubation of the viral protein with the human proteins and their labeling with the respective fluorescent antibodies. 6) MITOMI was then performed by actuation of the "button membrane" facilitating trapping surface-bound complexes while expelling any solution phase molecules. The "sandwich valves" were opened followed by a brief wash to remove untrapped unbound material. 7) The device was scanned by an array scanner. Trapped viral protein and surface bound human proteins were detected. The ratio of bound viral protein to expressed human protein was calculated for each data point by measuring the median signal of Cy3 to median signal of FITC (represented by ●).

(**c**) Surface patterning. 1) Accessible surface area was derivatized by flowing a solution of biotinylated BSA ( ) through all flow channels. 2) A Neutravidin solution ( ) was loaded. 3) The "button" membrane was activated. 4) All remaining accessible surface area except for a circular area of 60 µm masked by the button was passivated with biotinylated solution ( ). 5) The "button" membrane was opened. 6) A solution of biotinylated-anti-his antibodies ( ) was loaded allowing specific functionalization of the previously masked circular area 7) *In vitro* expressed human protein ( ) bound to the biotinylated-antibodies coating the discrete circular area. Each of the described steps was followed by a PBS wash.

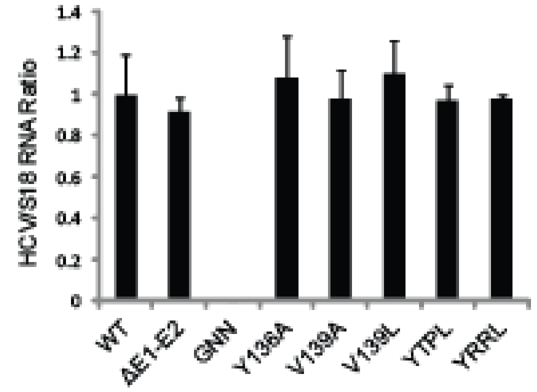


**Figure S2. Core's YXXΦ mutations do not affect HCV RNA replication by qRT-PCR assays.** HCV RNA replication, by qRT-PCR in Huh-7.5 cells 72 hr following electroporation with J6/JFH(p7-Rluc2A) harboring the corresponding core's YXXΦ mutations relative to WT control. ΔE1-E2 is an assembly defective control. GNN is a replication defective polymerase mutant. Means and s.d. (error bars) of results from two independent experiments in triplicates are shown.

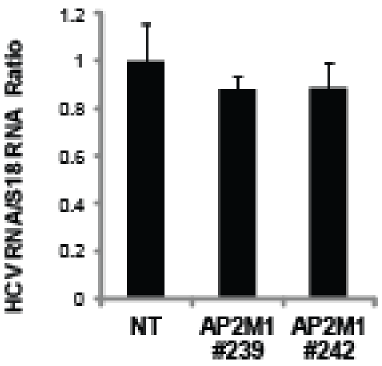


**Figure S3. AP2M1 depletion has no effect on HCV RNA replication by qRT-PCR assays.** HCV RNA replication, by qRT-PCR in Huh-7.5 cells harboring the corresponding shRNAs 72 hr following electroporation with J6/JFH(p7-Rluc2A) relative to NT control. Means and s.d. (error bars) of results from two independent experiments in triplicates are shown.

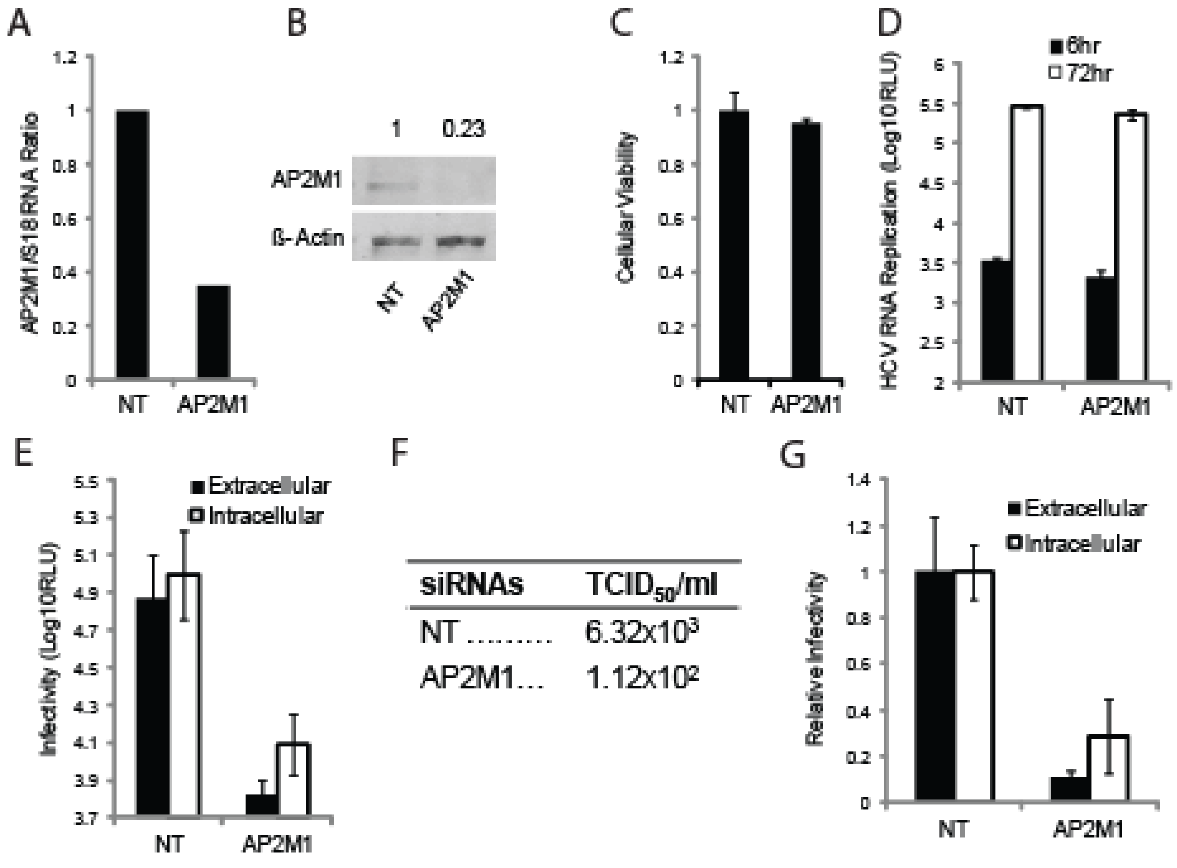


**Figure S4. Transient depletion of AP2M1 by pooled siRNAs inhibits HCV assembly.** A. AP2M1/S18 RNA ratio measured by qRT-PCR in Huh-7.5 cells transfected with a pool of four siRNAs (ON-TARGETplus SMARTpools, Dharmacon) targeting AP2M1 or a pool of non-targeting (NT) sequences at 48 hr posttransfection relative to NT contols. B. AP2M1 protein levels by quantitative Western analysis in cells 48 hr posttransfection with the corresponding pooled siRNAs. Numbers represent AP2M1 to actin protein ratio relative to the NT control. C. Cellular viability by alamarBlue assays 48 hr post siRNAs transfections relative to NT control. D. Cells were electroporated with J6/JFH(p7-Rluc2A) at 48 hr following transfection with the indicated pooled siRNAs. HCV RNA replication in these cells by luciferase assays at 6hr (black) and 72hr (white) postelectroporation. E. Extracellular (black) and intracellular (white) infectivity measured in naive Huh-7.5 cells infected with supernatants or clarified cell lysates derived from electroporated cells harboring the indicated siRNAs by luciferase assays, respectively. F. Infectious virus production measured by limiting dilution assays. G. Extracellular (black) and intracellular (white) infectivity measured by focus formation assays in naive Huh-7.5 cells infected with supernatants or clarified cell lysates derived from Huh-7.5 cells transiently depleted for AP2M1 by siRNAs and infected with culture grown J6/JFH virus (titer: $1.2x10^5$ $TCID_{50}$/ml). Results are relative to NT controls. Means ± s.d. (error bars) of results from at least two independent experiments are shown. RLU is relative light units. $TCID_{50}$ is 50% tissue culture infectious dose.

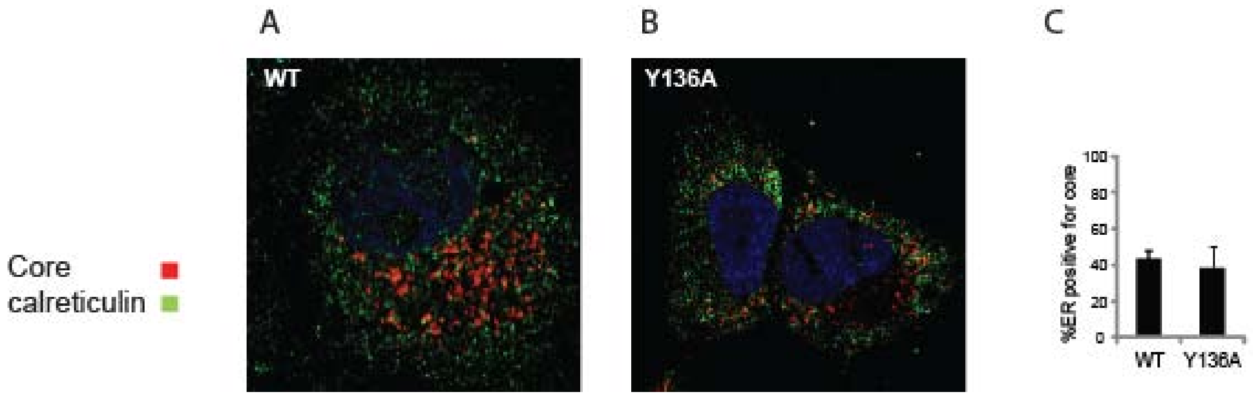


**Figure S5. Y136A core mutation does not seem to affect core localization to ER membranes in HCV infected cells.** A quantitative confocal immunofluorescence (IF) analysis for localization of core to the ER membrane in Huh-7.5 cells infected with culture grown HCV. A and B are representative merged images of core (red) and the ER marker, calreticulin (green), demonstrating a comparable partial localization of core to the ER membrane in Huh-7.5 cells infected with WT virus (A) or with virus harboring the Y136A core mutation (B). Representative images at x60 magnification are shown. C. Colocalization analysis of Z stacks using Manders' coefficients (with a higher value representing more colocalization). Values indicate mean M2 values represented as percent colocalization (the fraction of green intensity that coincides with red intensity ± s.d. (error bars); n = 10-15.

**Table S1. Antibodies used in this study**

| Antibodies | Source |
|---|---|
| Biotinylated anti-penta-his | Qiagen |
| FITC-conjugated anti-V5 | Invitrogen |
| Phycoerythrin-conjugated anti-T7 tag | Abcam |
| Rabbit Anti-AAK1 | Abcam |
| Rabbit anti-AP2M1 | Santa Cruz Biotechnology |
| Goat anti-AP2M1 | Santa Cruz Biotechnology |
| Rabbit anti-phospho-AP2M1 (T156) | Cell signaling |
| Rabbit anti-TGN46 | Abcam |
| Rabbit anti-calreticulin | Enzo Life Sciences |
| Mouse anti-GAK | MBL international corporation |
| Mouse anti-core | Austral Biologicals |
| Human anti-E2 (CBH5) | Dr. Steven K Foung |
| Mouse anti-actin | Sigma |
| HRP-conjugated anti-mouse IgG | Cell signaling |
| HRP-conjugated anti-rabbit IgG | Cell signaling |

## Table S2. Compounds used in this study

| Compound | Source |
|---|---|
| PKC-412 | Sigma |
| Sunitinib malate | Sigma |
| Erlotinib | LC Laboratories |
| Calyculin A | Cell Signaling |

## Table S3. RNAi used in this study

| **RNAi type** | **Target** | **Catalogue#** | **Source** | **Sequence** |
|---|---|---|---|---|
| ON-TARGETplus SMARTpools | AP2M1 | LQ-008170-00-0002 | Dharmacon | |
| | AP2M1 | J-004233-05 | Dharmacon | GUUAAGCGGUCCAACAUUU |
| | AP2M1 | J-004233-06 | Dharmacon | GCGAGAGGGUAUCAAGUAU |
| | AP2M1 | J-004233-07 | Dharmacon | AGUUUGAGCUUAUGAGGUA |
| | AP2M1 | J-004233-08 | Dharmacon | GAACCGAAGCUGAACUACA |
| | Non-targeting | D-001810-10-05 | Dharmacon | Non Available |
| siRNAs | AAK1 | Ref #4 | Ambion | GGUGUGCAAGAGAGAAAUCtt |
| | GAK | Ref #5 | Dharmacon | AACGAAGGAACAGCUGAUUCA |
| | Non-targeting | D-001210-01-05 | Dharmacon | Non Available |
| MISSION shRNAs | AP2M1 | TRCN0000060238 (“238”) | Sigma | CCGGGTGGTCATCAAGTCCAACTTTCTCGA GAAAGTTGGACTTGATGACCACTTTTTG |
| | AP2M1 | TRCN0000060239 (“239”) | Sigma | CCGGCACCAGCTTCTTCCACGTTAACTCGA GTTAACGTGGAAGAAGCTGGTGTTTTTG |
| | AP2M1 | TRCN0000060241 (“241”) | Sigma | CCGGGCTGGATGAGATTCTAGACTTCTCGA GAAGTCTAGAATCTCATCCAGCTTTTTG |
| | AP2M1 | TRCN0000060242 (“242”) | Sigma | CCGGCATTTATGAAACTCGCTGCTACTCGAG TAGCAGCGAGTTTCATAAATGTTTTTG |
| | Non-targeting | SHC002V | Sigma | Non Available |

**Table S4. Taqman probes and primers used in this study**

| Primer name | Sequence or assay catalogue # |
|---|---|
| HCV Forward | CTTCACGCAGAAAGCGTCTA |
| HCV Reverse | CAAGCACCCTATCAGGCAGT |
| HCV Taqman MGB probe | [6FAM]-TATGAGTGTCGTGCAGCCTC-[MGB-NFQ] |
| AP2M1 | HS01037584_m1 |
| GAK | HS00178347_m1 |
| AAK1 | HS00208618_m1 |
| S18 | hs999999_m1 |

(All the reagents listed in this table were purchased from Applied Biosystems, Inc (ABI)).

**Supporting References:**

1. Ricotta D, Hansen J, Preiss C, Teichert D, HÃning S (2008) Characterization of a Protein Phosphatase 2A Holoenzyme That Dephosphorylates the Clathrin Adaptors AP-1 and AP-2. Journal of Biological Chemistry 283: 5510-5517.
2. Murray CL, Jones CT, Tassello J, Rice CM (2007) Alanine Scanning of the Hepatitis C Virus Core Protein Reveals Numerous Residues Essential for Production of Infectious Virus. J Virol 81: 10220-10231.
3. Lindenbach BD, Evans MJ, Syder AJ, Wolk B, Tellinghuisen TL, et al. (2005) Complete replication of hepatitis C virus in cell culture. Science 309: 623-626.
4. Henderson DM. & Conner SD (2007) A Novel AAK1 Splice Variant Functions at Multiple Steps of the Endocytic Pathway. Molecular Biology of the Cell 18: 2698-2706.
5. Kametaka S, et al. (2007) Canonical Interaction of Cyclin G associated Kinase with Adaptor Protein 1 Regulates Lysosomal Enzyme Sorting. Molecular Biology of the Cell 18: 2991-3001.
